# FLIP the Switch: Regulation of Apoptosis and Necroptosis by cFLIP

**DOI:** 10.3390/ijms161226232

**Published:** 2015-12-18

**Authors:** Yuichi Tsuchiya, Osamu Nakabayashi, Hiroyasu Nakano

**Affiliations:** Department of Biochemistry, Toho University School of Medicine, Tokyo 143-8540, Japan; necozame@med.toho-u.ac.jp (O.N.); hiroyasu.nakano@med.toho-u.ac.jp (H.N.)

**Keywords:** apoptosis, caspase-8, cFLIP, necroptosis, TNF-α, ubiquitin-proteasome system

## Abstract

cFLIP (cellular FLICE-like inhibitory protein) is structurally related to caspase-8 but lacks proteolytic activity due to multiple amino acid substitutions of catalytically important residues. cFLIP protein is evolutionarily conserved and expressed as three functionally different isoforms in humans (cFLIP_L_, cFLIP_S_, and cFLIP_R_). cFLIP controls not only the classical death receptor-mediated extrinsic apoptosis pathway, but also the non-conventional pattern recognition receptor-dependent apoptotic pathway. In addition, cFLIP regulates the formation of the death receptor-independent apoptotic platform named the ripoptosome. Moreover, recent studies have revealed that cFLIP is also involved in a non-apoptotic cell death pathway known as programmed necrosis or necroptosis. These functions of cFLIP are strictly controlled in an isoform-, concentration- and tissue-specific manner, and the ubiquitin-proteasome system plays an important role in regulating the stability of cFLIP. In this review, we summarize the current scientific findings from biochemical analyses, cell biological studies, mathematical modeling, and gene-manipulated mice models to illustrate the critical role of cFLIP as a switch to determine the destiny of cells among survival, apoptosis, and necroptosis.

## 1. Introduction

The homeostasis of our tissues, organs, and whole body is maintained by the continuous flow of birth, growth, differentiation, and death of cells. Cell death is therefore the indispensable component of our life, and several sophisticated cell death-inducing machineries are installed in the genomes of multicellular organisms. Apoptosis is the most popular pathway to eliminate unnecessary or harmful cells. In addition, recent scientific advances have revealed that other regulated cell death pathways, including programmed necrosis also known as necroptosis, mitochondrial permeability transition-driven regulated cell death, pyroptosis, and ferroptosis, play important roles in maintaining tissue homeostasis under normal and pathological conditions [1].

**Figure 1:**
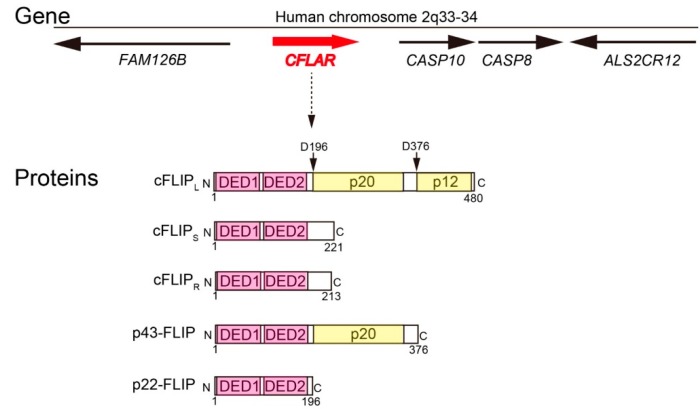
The structures of gene and proteins for human cFLIP. For gene structure, the red arrow (*CFLAR* gene) and black arrows (the nearby genes) indicate the positions and directions of genes present on human chromosome 2q33-34. For protein structures, light magenta and light yellow boxes indicate DEDs and caspase-8-like domains, respectively. The numbers below the boxes indicate amino acid residues, and arrows above the boxes indicate the caspase-8-mediated cleavage sites.

One physiological function of apoptosis is to kill and remove virus-infected cells in order to protect the hosts from viral propagation. To escape from the host’s protective machinery, some viruses express anti-apoptotic proteins to prevent the host cells from apoptotic cell death. In 1997, Thome *et al.* [2] identified viral FLICE-inhibitory proteins (vFLIPs), which contained two death effector domains (DEDs) and interfered with apoptosis signaling through death receptors. As vFLIPs were highly similar to the N-terminus of procaspase-8 (also known as FLICE, MACH or Mch-5), it was assumed that these viral genes might be derived from host genes. As expected, Irmler *et al.* [3] identified a highly related gene in human genome and named this as *CFLAR* (CASP8 and FADD-like apoptosis regulator). *CFLAR* gene is located on human chromosome 2q33-34 adjacent to genes encoding caspase-8 and caspase-10, suggesting that these three genes were generated by ancient gene duplication (Figure 1).

Human *CFLAR* gene is composed of 14 exons, and multiple mRNAs are produced via alternative splicing. The protein product of *CFLAR* gene, named as cellular FLIP or cFLIP, is expressed as three major isoforms in humans. cFLIP_L_ is a 55 kDa protein containing N-terminal two DEDs and C-terminal caspase-like domain. Although this domain organization is highly similar to that of procaspase-8, the catalytically important amino acid residues are not conserved in cFLIP_L_. Therefore, cFLIP_L_ does not possess caspase-like proteolytic activity by itself. cFLIP_S_ is a 27 kDa protein composed of only two DEDs without caspase-like domain, but is absent in mice due to the lack of corresponding exon in mice genome. These proteins were also independently identified and named as CASH, CASP8AP1, CLARP, Casper, FLAME, FLIP, I-FLICE, MRIT, or usurpin, suggesting that the discovery of cFLIP had a significant impact on the field of cell death research [4,5,6,7,8,9,10]. Another short form protein of 25 kDa, named as cFLIP_R_, is specifically expressed in some cell lines such as Raji and SKW6.4 and in human primary T cells [11,12]. Ueffing *et al.* [13] reported that a single nucleotide polymorphism, named as rs10190751, determines whether human *CFLAR* gene produces cFLIP_S_ or cFLIP_R_. All isoforms are currently supposed to form heterodimers with caspase-8 via DED–DED interaction. In addition, truncated cFLIP fragments named as p43-FLIP and p22-FLIP are generated by caspase-8-mediated cleavages after Asp376 and Asp196, respectively. The gene encoding cFLIP is evolutionarily conserved in vertebrates [14], and both cFLIP_L_ and cFLIP_R_ are also expressed in mice. Asp376 of human cFLIP_L_ is conserved in mouse cFLIP_L_ (Asp377), whereas Asp196 is only present in human cFLIP_L_. Therefore, p22-FLIP is produced by caspase-8-mediated cleavage only in humans.

Extensive analyses have revealed that cFLIP controls not only the classical death receptor-mediated extrinsic apoptosis pathway, but also the non-conventional pattern recognition receptor-dependent apoptotic pathway. In addition, cFLIP regulates the formation of death receptor-independent apoptosis platform named as ripoptosome. Moreover, recent finding have also indicated the involvement of cFLIP during another cell death pathway named as necroptosis. Therefore, cFLIP exerts critical functions to determine the cellular fate between survival and death in a highly regulated manner.

In this review, we will focus on three topics of the physiological roles of cFLIP as follows: (1) molecular functions of cFLIP in death receptor-mediated apoptosis pathway, ripoptosome formation, and necroptosis; (2) quantitative regulation of cFLIP by the ubiquitin-proteasome system; and (3) physiological roles of cFLIP to maintain tissue and systemic homeostasis in mammals. We encourage the readers to also refer to our complementary review [15]. The cellular functions as well as transcriptional and post-translational regulation of cFLIP are also extensively reviewed by Safa [16].

## 2. Molecular Functions of cFLIP in Death Receptor-Mediated Apoptosis Pathway, Ripoptosome Formation, and Necroptosis

### 2.1. Molecular Function of cFLIP in Death Receptor-Dependent Apoptosis Pathway

The death receptor-mediated extrinsic apoptosis pathway is initiated when the extracellular tumor necrosis factor (TNF) superfamily death ligands including TNF-α, Fas ligand/CD95L, and TNF-related apoptosis-inducing ligand (TRAIL) bind to specific cell surface death receptors. These ligand–receptor interactions induce the oligomerization of receptor subunits, association of adaptor proteins including Fas-associated death domain (FADD) or TNF receptor-associated death domain (TRADD) via the interaction between death domain (DD) of receptors and adaptors. Next, the DED of FADD binds to DED of procaspase-8/10 to form death-inducing signaling complex (DISC). Local concentration of procaspase-8/10 at DISC leads to the formation of procaspase-8/10 homodimer and subsequent self-processing to generate active caspase-8/10. Fully processed active caspase-8/10 is then released from DISC and activates downstream effector caspases. Caspase-10 appears functionally similar to caspase-8, but the absence of caspase-10 in rodents suggests that caspase-8 is the major initiator in extrinsic apoptosis pathway, and we therefore discuss only caspase-8 in this review. As cFLIP is highly similar to procaspase-8 but lacks catalytic activity by itself, one may easily suppose that all cFLIP isoforms inhibit the processing of procaspase-8 through heterodimerization. However, the initial characterization of cFLIP by cellular overexpression studies gave conflicting results with regard to apoptosis regulation [3,4,5,6,7,8,9,10,17,18,19]. Extensive biochemical analysis revealed that cFLIP_L_ was in fact a more potent activator of procaspase-8 than procaspase-8 itself [20], and forced dimerization experiments suggested that cFLIP_L_ was able to activate procaspase-8 without interdomain cleavage and altered its substrate specificity [21]. Yu *et al.* [22] found that p43-FLIP, generated by procaspase-8-mediated cleavage, showed enhanced heterodimerization with procaspase-8 than noncleaved cFLIP_L_. Therefore, cFLIP_L_-mediated caspase-8 regulation is more complex than initially suggested. In contrast, it is believed that both cFLIP_S_ and cFLIP_R_ simply block procaspase-8 activation through heterodimerization.

**Figure 2:**
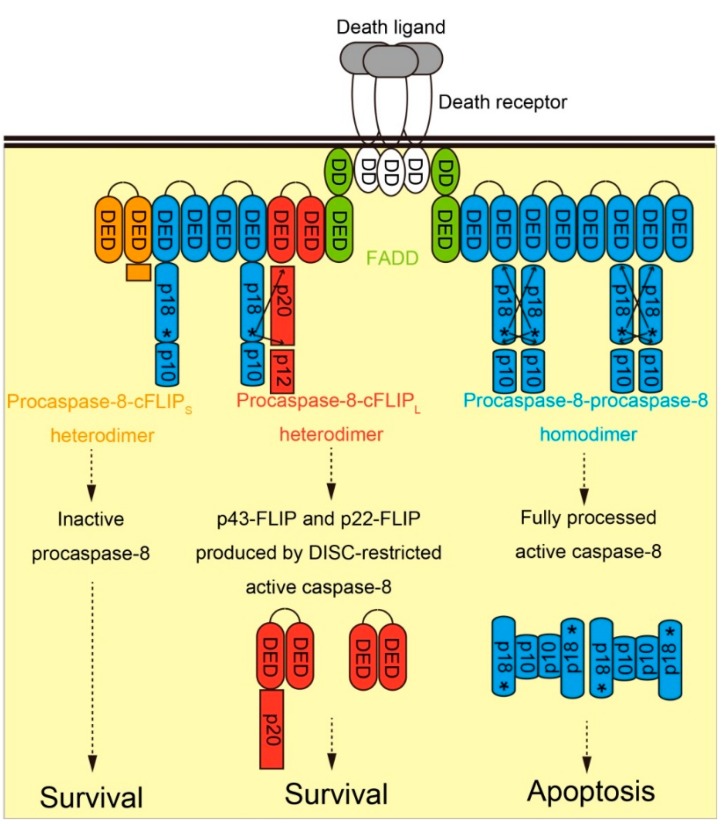
Functional role of cFLIP during classical death receptor-mediated extrinsic apoptosis pathway. Upon stimulation by death ligand (gray), death receptor (white) forms trimer and activated. Adaptor protein FADD (green) then binds to activated death receptor via DD-DD interaction. Subsequently, DED-containing proteins including procaspase-8 (blue), cFLIP_L_ (red), and cFLIP_S_ (orange) are recruited to death receptor-bound FADD via DED-DED interaction, thereby forming DISC. Fully processed active caspase-8, generated by procaspase-8 homodimerization, activates effector caspases and induces apoptosis. Procaspase-8-cFLIP_L_ heterodimerization results in the production of p43-FLIP and p22-FLIP but does not process procaspase-8, leading to cellular survival. In contrast, procaspase-8-cFLIP_S_ heterodimerization inhibits the activation of procaspase-8 and prevents apoptosis.

Recent technical advances of quantitative mass spectrometric analysis enabled us to estimate not only the presence, but also the amount of molecules in a given protein complex. Using this technique, Dickens *et al.* [23] estimated the stoichiometry of TRAIL-induced DISC, whereas Schleich *et al.* [24] analyzed the stoichiometry of CD95L-induced DISC. Both researchers found that DISC contained several-fold more procaspase-8 than FADD, and that the amount of cFLIP in native DISC was very low. These results suggested that multiple procaspase-8 molecules could bind to single FADD molecule or cFLIP. Previous studies indicated that DEDs from procaspase-8, FADD, and cFLIP formed filament-like structure known as “death-effector filaments” when overexpressed in cells [25], suggesting that procaspase-8 in DISC might form chain-like structure through DED–DED interaction. In contrast, Majkut *et al.* [26] carried out quantitative Western blotting to find that the ratio of (procaspase-8 + cFLIP):FADD in DR5 agonistic antibody-induced DISC was 2:1 at most, arguing against the DED chain model. However, the stoichiometry may be dependent on cell types, death receptor-death ligand specificities, and the strength of death signals. Majkut *et al.* [26] further combined site-directed mutagenesis and molecular modeling to propose a two-step DISC model. According to their model, homodimerization of procaspase-8 at DISC produces fully processed caspase-8 and efficiently induces apoptosis. cFLIP_L_ incorporated in the chain can form heterodimer with procaspase-8 and activate it in the absence of interdomain processing. However, cFLIP_L_-activated procaspase-8 is bound to DISC via DED-DED interaction, and cleaves only limited substrates around DISC. This model may explain why cFLIP_L_ can inhibit apoptosis while possessing the biochemical ability to activate procaspase-8. Kallenberger *et al.* [27] also used cell compartment-specific fluorescent probes to establish a mathematical model of procaspase-8 activation kinetics. They concluded that procaspase-8 was first cleaved at prodomain in an intra-dimeric manner within a single DISC. Subsequently, the association of multiple DISCs induced the cleavage of procaspase-8 at enzymatic domain in an inter-dimeric manner, followed by the cytoplasmic release of fully processed caspase-8. In contrast, cFLIP_S_ and cFLIP_R_ incorporated in the chain bind to but cannot activate procaspase-8. Similarly, Schleich *et al.* [28] very recently reported that the N-terminal prodomain of procaspase-8, generated by the complete processing of procaspase-8 and structurally related to cFLIP_S/R_, remained in DISC and constituted a negative feedback loop to terminate procaspase-8 activation. These short isoforms were initially believed to maintain cell survival by simply inhibiting apoptosis (Figure 2). However, in some situations, the inactive procaspase-8-cFLIP_S/R_ complex can initiate an alternative non-apoptotic cell death program termed as necroptosis, which will be described below.

### 2.2. Molecular Function of cFLIP in Ripoptosome Formation and Necroptosis

**Figure 3:**
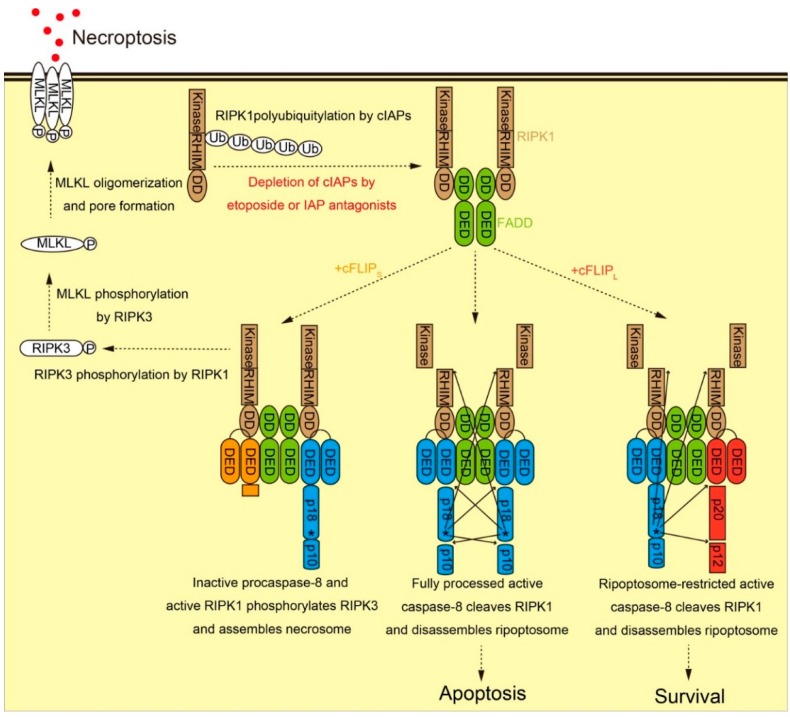
Functional role of cFLIP during ripoptosome formation. RIPK1 (brown) is composed of catalytic kinase domain, RIP homotypic interaction motif (RHIM), and DD. Upon RIPK1 activation by genotoxic stress, DD of RIPK1 is exposed and binds to DD of FADD (green). Subsequently, DED-containing proteins including procaspase-8 (blue), cFLIP_L_ (red), and cFLIP_S_ (orange) are recruited to RIPK1-bound FADD via DED-DED interaction, thereby forming ripoptosome. Fully processed active caspase-8, generated by procaspase-8 homodimerization, activates effector caspases, cleaves RIPK1, disassembles ripoptosome, and induces apoptosis. Procaspase-8-cFLIP_L_ heterodimer produces p43-FLIP and p22-FLIP, cleaves RIPK1, disassembles ripoptosome, but does not process procaspase-8, leading to cellular survival. In contrast, procaspase-8-cFLIP_S_ heterodimer fails to cleave RIPK1. This leads to the assembly of necrosome composed of RIPK1-RIPK3-MLKL and the execution of necroptosis.

Under specific conditions, cells can activate caspase-8 in the absence of extrinsic death ligand–death receptor interactions. Receptor interacting protein kinase 1 (RIPK1), also known as RIP1, was originally identified as a protein which interacted with TNF receptor 1 signaling complex [29,30]. Recent explosive scientific advances have revealed the critical roles of RIPK1 in cell fate regulation among survival, apoptosis, and necroptosis. RIPK1 itself is also functionally regulated through phosphorylation and ubiquitylation. Cellular inhibitors of apoptosis proteins (cIAPs) are E3 ubiquitin ligases known to ubiquitylate RIPK1 as well as themselves [31]. To examine whether the elimination of cIAPs via self-ubiquitylation and proteasome-mediated degradation could affect cell death programs, Tenev *et al.* [32] used a cytotoxic drug etoposide to induce genotoxic stress-induced cell death. Feoktistova *et al.* [33] employed an IAP antagonist, also known as Smac mimetic, in combination with dsRNA poly I:C to induce Toll-like receptor 3 (TLR3)-mediated cell death. Both authors observed the spontaneous, death receptor-independent formation of ripoptosome, a 2 MDa complex containing RIPK1, FADD, caspase-8 and cFLIP (Figure 3).

According to the currently proposed model [34], procaspase-8 incorporated in ripoptosome is activated and fully processed through homodimerization, cleaves and inactivates RIPK1, and disassembles ripoptosome. As a consequence, only caspase-8-mediated apoptosis is executed. When cFLIP_L_ is incorporated in ripoptosome, it activates procaspase-8 through heterodimerization. However, cFLIP_L_-activated procaspase-8 is able to cleave limited substrates and inactivate RIPK1, but becomes inactive again after ripoptosome disassembly. As a consequence, cFLIP_L_ blocks apoptosis and maintains cell survival.

In contrast, cFLIP_S_ promotes ripoptosome assembly but cannot activate procaspase-8 to inactivate RIPK1. Under these conditions, cells undergo another type of cell death known as necroptosis. Necroptosis is clearly distinct from accidental necrosis, as it is physiologically regulated by specific proteins including RIPK1, receptor-interacting protein kinase 3 (RIPK3, also known as RIP3), and mixed lineage kinase-like protein (MLKL). These proteins are the main components of necroptosis-inducing complex known as necrosome. In the current scenario, RIPK1 phosphorylates and activates RIPK3, and activated RIPK3 then phosphorylates MLKL. Phosphorylated MLKL forms oligomer via N-terminal four helix bundles domain, translocates to plasma membrane, and induces necrotic cell death by forming pores on the plasma membrane [35]. The activation of necroptosis pathway requires the presence of functional RIPK1-RIPK3-MLKL axis, as He *et al.* [36] reported that several cell lines lacking RIPK3 expression were resistant to necroptosis. Feoktistova *et al.* [37] also reported that necroptosis promoted by the elimination of IAPs and the overexpression of MC159 vFLIP was dependent on the kinase activity of RIPK3. Inhibition of RIPK3 kinase activity by either chemical compounds or active site D161N mutation (but not other inactive mutants) blocked necroptosis, but unexpectedly induced apoptosis via the formation of ripoptosome-like platform. Unlike TNF-α-induced apoptosis, RIPK3 inhibitor-induced apoptosis was enhanced by cFLIP_L_ in mouse embryonic fibroblasts [38]. These results indicate that the functional RIPK1-RIPK3-MLKL axis and the balance of cFLIP isoforms are both critical determinants of cell fate switching among survival, apoptosis, and necroptosis. However, it is still not clear whether cFLIP_S_ and vFLIP actively promotes necroptosis execution, or simply fail to inhibit necroptosis due to their inability to block RIPK1-RIPK3-MLKL axis.

cFLIP is also involved in the formation of other death-inducing protein complexes. Day *et al.* [39] identified an apoptotic inhibitory complex comprised of DR5-, FADD-, Caspase-8-, and cFLIP_L_.in MCF-7 breast cancer cells. They found that the removal of cFLIP_L_ from this complex resulted in the spontaneous ligand-independent apoptosis, although it was not clear whether RIPK1 was involved in this process. Estornes *et al.* [40] reported the dsRNA-induced formation of an atypical death complex composed of TLR3, TIR domain-containing adaptor inducing interferon-β (TRIF), and caspase-8. In contrast with ripoptosome, this TLR3-dependent complex required RIPK1 but not FADD to activate procaspase-8. These results suggest that multiple ripoptosome-like apoptosis-inducing complexes can be formed by different death-inducing stimuli. 

## 3. Quantitative Regulation of cFLIP by Ubiquitin-Proteasome System

### 3.1. cFLIP Concentration as a Critical Parameter for Cell Fate Determination Revealed by Mathematical Modeling

The endogenous concentration of cFLIP in cells is generally low compared to that of procaspase-8. Mathematical modeling studies have revealed that the ratio between procaspase-8 and cFLIP is a critical parameter for cell fate determination by death receptor signaling. Bentele *et al.* [41] developed a mathematical modeling framework model of CD95L-mediated apoptosis and found that the concentration of cFLIP_L_ determined the threshold ligand concentration. Moreover, Lavrik *et al.* [42,43] experimentally revealed that the low concentrations of CD95L below the threshold resulted in extracellular signal-regulated kinase (ERK) activation and transduction of survival signaling. Accordingly, cFLIP_L_ could either accelerate or slow down cell death in a CD95L and cFLIP_L_ dose-dependent manner, whereas cFLIP_S_ and cFLIP_R_ only inhibited procaspase-8 activation [44]. Computational simulation by Han *et al.* [45] also predicted that cFLIP_L_ established bistability in caspase-8 and caspase-3 activation. In addition, Neumann *et al.* [46] introduced an integrated kinetic mathematical model for CD95L-mediated apoptotic and NF-κB signaling. They concluded that cFLIP_L_ recruitment kinetics to DISC and subsequent generation of p43-FLIP was the critical parameter, and that a subtle balance between cFLIP_L_ and procaspase-8 determined life/death decision in a nonlinear manner. According to their model, the precise quantitative regulation of cFLIP is a critical factor to determine the death ligand sensitivity and cell fate determination. The endogenous expression levels of cFLIP and caspase-8 are highly variable among cell lines, which may explain the different results in the previously published cellular studies [47].

### 3.2. Regulation of cFLIP Concentration by Proteolysis

The next question is: how is the concentration of cFLIP protein regulated? The amount of a given protein may be regulated transcriptionally, translationally, and post-translationally. Among these, protein degradation is the quickest way to change its concentration. In this section, we specifically focus on the post-translational regulation of cFLIP by proteolysis. All isoforms of cFLIP are reported to be unstable proteins with rapid turnover, and a mathematical modeling study by Toivonen *et al.* [48] indicated cFLIP turnover as a key determinant of death receptor responses. Previous literatures have suggested that cFLIP is mainly degraded via the ubiquitin-proteasome pathway. A quantitative modeling study by Roux *et al.* [49] also suggested that the inhibition of proteasome significantly affected cFLIP concentration and death signal sensitivity in an isoform-specific manner. As most substrate proteins require prior polyubiquitylation to be degraded by proteasome, cFLIP polyubiquitylation is thought to play critical roles in regulating cell fate. However, the E3 ubiquitin ligases responsible for the polyubiquitylation of cFLIP may not be a single one. Rather, it appears that various E3 ubiquitin ligases recognize and polyubiquitylate cFLIP in an isoform-, cell type-, context-, or signal-dependent manner. Here, we summarize the examples of these E3 ubiquitin ligases for cFLIP and discuss their physiological relevance (Table 1).

**Table 1:** Candidate E3 ubiquitin ligases for cFLIP polyubiquitylation.

E3 ubiquitin Ligase	Type	Substrate	References
Itch	HECT	cFLIP_L_	[50,51,52,53,54,55,56,57,58,59,60]
Cbl	RING	cFLIP_S_	[50,61]
Cbl-b	RING	cFLIP_L_	[62]
Mind bomb1	RING	cFLIP_L_	[51]
TRAF7	RING	cFLIP_L_	[52]
CHIP	U-box	cFLIP_L_	[53]

### 3.3. Itch as an E3 Ubiquitin Ligase for cFLIP Polyubiquitylation

Chang *et al.* [54] identified that Itch, an E3 ubiquitin ligase also known as AIP4, was required for cFLIP_L_ degradation in a Jun N-terminal kinase 1 (JNK1)-dependent manner during TNF-α-dependent cell death. We previously reported that cFLIP_L_ directly bound to MAP kinase kinase 7 (MKK7) and inhibited JNK pathway [55], and that the downregulation of cFLIP induced JNK activation and the accumulation of reactive oxygen species in tumor cells [56]. Therefore, the mutual regulation between cFLIP and JNK-Itch pathway might be critically important to determine the destiny of cells. Murata *et al.* [57] reported that the interaction between cFLIP_L_ and Itch was blocked in *Trypanosoma cruzi*-infected HeLa cells, suggesting that the inhibition of cFLIP_L_ degradation was the parasite’s survival strategy to prevent host cell apoptosis. Demange *et al.* [58] reported that TG interacting factor (TGIF), a homeodomain protein, promoted TNF-α-dependent apoptosis by increasing the accessibility between cFLIP_L_ and Itch. Panner *et al.* [59,60] reported that Itch itself was ubiquitylated in an Akt-dependent manner in glioblastoma multiform. The inhibition of Itch ubiquitylation, either by the depletion of PTEN or by the overexpression of ubiquitin-specific protease 8 (USP8), also led to cFLIP_S_ ubiquitylation. Similarly, Haimerl *et al.* [63] reported that the TNF-α-mediated downregulation of USP2, another ubiquitin-specific protease for polyubiquitylated Itch, stabilized both cFLIP_L_ and cFLIP_S_ in hepatocytes.

Itch is also reported to be regulated by p53 tumor suppressor protein. Abedini *et al.* [64,65] found that cisplatin treatment induced the formation of Itch-p53-cFLIP complex and subsequent cFLIP polyubiquitylation. The same group recently showed that gelsolin, a regulator protein of actin dynamics, was also involved in the regulation of Itch-p53-cFLIP and conferred chemoresistance against cisplatin in gynecologic cancers [66]. It was also shown that ataxia telangiectasia mutated (ATM), a protein kinase required for DNA damage checkpoint, activated Itch through Ser161 phosphorylation and induced cFLIP_L_ degradation [67]. These results indicate that cFLIP is regulated not only by TNF-α-mediated extrinsic signal pathway, but also by DNA damage-induced intrinsic signal pathway. In addition, cystatin B, an endogenous inhibitor protein of lysosomal cathepsins, reduced the amount of Itch and increased the level of cFLIP_L_ in melanoma cells [68].

However, several studies have identified Itch-independent polyubiquitylation of cFLIP isoforms. Shi *et al.* [69] reported that the TNF-α-induced degradation of cFLIP_L_ in macrophages was mediated by PI3 kinase-Akt signaling pathway but independent of JNK-Itch. Sánchez-Pérez *et al.* [70,71] found that mitotic arrest-induced proteasomal degradation of cFLIP by microtubule-interfering agents was independent of Itch and sensitized cells to TRAIL-induced apoptosis in breast cancer cells. Moreover, Yerbes and Lόpez-Rivas also reported that a histone deacetylase inhibitor suberoylanilide hydroxamic acid induced the Itch-independent proteasomal degradation of both cFLIP_L_ and cFLIP_S_ and sensitized breast tumor cells to TRAIL [72]. These results clearly indicate that Itch is not the sole E3 ubiquitin ligase responsible for cFLIP polyubiquitylation.

### 3.4. Other Candidate E3 Ubiquitin Ligases for cFLIP Polyubiquitylation

Other E3 ubiquitin ligases were also identified in different cellular contexts. Kundu *et al.* [50] observed Cbl-dependent polyubiquitylation and proteasomal degradation of cFLIPs during *Mycobacterium tuberculosis*-induced apoptosis of macrophages. Zhao *et al.* [61] also showed that Cbl-dependent degradation of cFLIP_S_ was accelerated by the inhibition of mTOR complex 2 in non-small cell lung carcinoma cells. In contrast, Zhang *et al.* [62] showed that Cbl-b was involved in the arsenic trioxide-induced degradation of cFLIP_L_ in leukemic and gastric cancer cells. Moreover, Zhang and Gallaghar reported that Mind bomb 1, a multi-domain E3 ubiquitin ligase involved in Notch signaling, induced cFLIP_L_ degradation when overexpressed in 293T and HeLa cells [51]. Scudiero *et al.* [52] reported that TNF receptor-associated factor 7 (TRAF7), another E3 ubiquitin ligase, induced Lys29-, Lys33-, Lys48, and Lys63-linked polyubiquitylation of cFLIP_L_. Interestingly, unconventional Lys29-linked polyubiquitylation promoted lysosomal degradation of cFLIP_L_ in addition to proteasome-mediated degradation. Wang *et al.* [53] reported that C-terminus of HSP70-interacting protein (CHIP), another E3 ubiquitin ligase, was involved in the downregulation of cFLIP_L_ induced by HSP90 inhibition in lung cancer cells.

### 3.5. Post-Translational Modifications Regulating cFLIP Stability

A number of studies have indicated that the stability of cFLIP is regulated by phosphorylation, and some researchers have identified the phosphorylation sites and responsible kinases. Kaunisto *et al.* [73] found that Ser193, present in all cFLIP isoforms, was phosphorylated by protein kinase C. They showed that phospho-mimetic mutation of Ser193Asp prolonged the half-lives of cFLIP_S_ and cFLIP_R_ but not cFLIP_L_. Wilkie-Grantham *et al.* [74] reported the generation of reactive oxygen species induced the phosphorylation of cFLIP_L_ at Thr166, which was required for the subsequent polyubiquitylation at Lys167. In addition, *S*-nitrosylation was also reported to affect the stability of cFLIP. Chanvorachote *et al.* [75] found that the generation of nitric oxide induced *S*-nitrosylation of cFLIP_L_ at Cys254 and Cys259, which inhibited the polyubiquitylation of cFLIP_L_ and protected cells from CD95-induced apoptosis. cFLIP stability might be regulated by other types of post-translational modifications, including acetylation, methylation or oxidation. Further genetic and proteomic approaches will reveal more information concerning the post-translational modifications of cFLIP.

### 3.6. Physiological Significance of cFLIP Polyubiquitylation

One may wonder why cFLIP can be ubiquitylated by so many E3 ubiquitin ligases. One possibility is that the cellular level of cFLIP is strictly regulated by both constitutive and signal-dependent pathways in a different manner. As discussed in the above section, the cellular level of cFLIP is a critical determinant of cell survival and death in the extrinsic apoptosis pathway. Therefore, the level of cFLIP may be differentially regulated between cell death-sensitive and cell death-resistant cells. Another important point is the intrinsic instability of cFLIP proteins. Endogenous cFLIP was reported to be downregulated by heat stress or “hyperthermia”, suggesting that cFLIP is prone to denaturation or aggregation [76,77,78,79]. Indeed, Ishioka *et al.* [80] reported that the overexpressed cFLIP_L_ formed aggregates in the cells and impaired the normal activity of the ubiquitin-proteasome system. Hence, we should be cautious in interpreting the results of cFLIP degradation from cellular overexpression experiments. It is necessary to discriminate whether the experimentally observed cFLIP polyubiquitylation is a physiologically regulated phenomenon, or is simply a response against misfolded proteins such as Lys63-linked polyubiquitylation for aggresome targeting [81].

## 4. Physiological Roles of cFLIP in Maintaining Tissue Homeostasis in Mammals

### 4.1. Embryonic Lethality of Cflip-Deficient Mice

Gene targeting in mice is one of the most effective ways to analyze the physiological functions of the gene of interest in mammals. However, the initial experiment revealed that the mice embryos lacking all isoforms of cFLIP from whole body, generated by disrupting the exon 1 of *Cflar* gene, could not survive beyond E10.5 [82]. Shibata *et al*. [83], very recently reported the *Cflip-l+46* mutant mice, which expressed cFLIP_L_ with C-terminal 46-amino acid extension due to a mutation at the stop codon (X482W). As this cFLIP_L_ mutant protein was polyubiquitylated by TRIM21 E3 ubiquitin ligase and rapidly degraded by proteasome, these mice also died at around E13.5. These results clearly showed the importance of cFLIP during normal mammalian development, but also prevented us from investigating the physiological functions of cFLIP in adult animals. Since then, various researchers including us have employed special techniques or strategies to overcome this embryonic lethal problem. Here, we briefly summarize the experimental results using double- and triple-knockout mice, tissue-specific conditional knockout mice, and isoform-specific transgenic mice (Table 2).

**Table 2:** Phenotypes of *cFLIP* gene-manipulated mice.

	Genotype/Organ	Phenotype	References
Whole body KO mice	*Cflip^−/−^*	Embryonic lethality	[82]
	*Cflip-l+46* mutant	Embryonic lethality	[83]
	*Cflip^−/−^Ripk3^−/−^*	Embryonic lethality	[84]
	*Cflip^−/−^Fadd^−/−^Ripk3^−/−^*	Normal development	[84]
Conditional KO mice	T cells	Increased cell death	[85,86,87,88]
	B cells	Increased cell death	[89,90]
	Myeloid lineage	Growth retardation, Splenomegaly	[91,92]
	Dendritic cells	Increased inflammation	[93,94]
	Liver	Increased liver failure	[95,96,97,98,99,100]
	Intestine	Increased cell death and inflammation	[95,101]
	Skin epidermis	Increased cell death and inflammation	[102,103]
Transgenic mice	cFLIP_R_ in *Cflip*-deficient T cells	Similar to *Cflip*-deficient T cells	[104,105]
	cFLIP_L_ in T cells	Increased survival	[106,107,108,109,110,111,112,113,114,115]
	cFLIP_S_ in T cells	Increased survival	[116,117]
	cFLIP_L_ in neuron	Increased survival	[118]
	cFLIP_L_ in thyroid	Better resolution from autoimmune disease	[119,120,121,122]
	cFLIP_L_ in heart	Reduced cardiac hypertrophy, prevention of cardiac remodeling	[123,124,125]
	cFLIP_L_ in eosinophil	Increased survival	[126]
	cFLIP_L_ in testis	Testis atrophy	[127]
	cFLIP_L_ in muscle	Muscle aging	[128]
	cFLIP_R_ in hematopoietic cells	Better bacterial clearance, increased autoimmune disease	[129,130]

### 4.2. Rescue of Cflip-Deficient Mice by Ablating Apoptosis and Necroptosis

Caspase-8 is the principal initiator caspase, and FADD is the essential adaptor protein for the death receptor-mediated extrinsic apoptosis pathway. Both *Casp8*-deficient and *Fadd*-deficient mice are embryonic lethal, indicating their indispensable survival functions for normal development. Oberst *et al.* [131] reported that the embryonic lethal phenotype of *Casp8*-deficent mice was rescued by the additional deletion of *Ripk3*, a central player of necroptosis, although these mice displayed a progressive lympho-accumulative disease. They further showed that the catalytically active caspase-8-cFLIP_L_ complex prevented RIPK3-dependent necroptosis without inducing apoptosis. Subsequently, the same group showed that both *Fadd^−/−^Ripk3^−/−^* double knockout mice and *Cflip*^−^*^/^*^−^*Fadd*^−^*^/^*^−^*Ripk3*^−^*^/^*^−^ triple knockout mice were viable in a similar manner to *Casp8*^−^*^/^*^−^*Ripk3*^−^*^/^*^−^ double knockout mice. However, the embryonic lethal phenotype of *Cflip*-deficient mice was not rescued by the additional deletion of *Ripk3* alone. These results suggest that, in the presence of RIPK3, the active caspase-8-cFLIP_L_ complex is necessary to prevent RIPK3-dependent necroptosis. In contrast, when RIPK3 is absent, cFLIP plays essential roles in blocking caspase-8- and FADD-dependent extrinsic apoptosis pathway [84,102]. Therefore, cFLIP_L_ is required for the inhibition of both apoptosis and necroptosis to maintain the normal development and survival of mice.

### 4.3. Conditional Knockout Mice Lacking cFLIP in T Cells

Chau *et al.* [85] analyzed *Cflip*-deficient T cells from chimeric mice generated by reconstituting *Rag1*^−/−^ blastocysts with *Cflip*-deficient embryonic stem cells. They found that *Cflip*-deficient T cells were defective in T cell receptor (TCR)-stimulated proliferation and cell survival, but TCR-induced ERK activation was not affected. Zhang and He generated *Cflip*-floxed mice in which exon 1 of *Cflar* gene was flanked by two *loxP* sites (described as *Cflip^F/F^* hereafter). They crossed *Cflip^F/F^* mice with the transgenic mice expressing *Cre* under *Lck* promoter to generate mice lacking *Cflip* only in T cells. These mice showed severely impaired T cell maturation at the single positive thymocyte stage due to increased apoptosis, although NF-κB and ERK signaling remained intact [86]. The same group showed that cFLIP also protected mature T cell from not only death receptor-mediated extrinsic apoptosis, but also from death induced by TCR engagement [87]. As an alternative approach, *Cflip* deletion was induced by tamoxifen administration in *Cflip^F/F^* mice expressing *ERT2-Cre* under *Rosa26* promoter. Using this system, He *et al.* [88] isolated mature T cells from these mice and deleted *Cflip* from these cells by the addition of tamoxifen *in vitro*. They found that *Cflip* protected resting mature T cells not only from extrinsic apoptosis pathway, but also from staurosporine-induced intrinsic apoptosis pathway.

### 4.4. Conditional Knockout Mice Lacking cFLIP in Other Lineages of Blood Cells

The physiological roles of cFLIP in B cells were also investigated by the expression of *Cre* under *Cd19* promoter in *Cflip^F/F^* mice. Zhang *et al.* [89] reported that *Cflip*-deficient B cells developed normally but were hypersensitive to Fas-induced apoptosis and resistant to Toll-like receptors (TLRs)- and B-cell receptor (BCR)-induced proliferation. Although NF-κB and ERK signaling was unaffected, p38 and JNK were aberrantly activated in *Cflip*-deficient B cells. Moreover, Coffey and Manser found that the *Cflip*-deficient B cells showed reduced recruitment into germinal center response [90].

Gordy *et al.* [91] expressed *Cre* under *Lyz2* promoter in *Cflip^F/F^* background to generate mice lacking cFLIP in myeloid lineage. Huang *et al.* [92] independently generated *Cflip*-floxed mice in which exons 2-3 of *Cflar* gene were flanked by *loxP* sequences, and they also generated myeloid-specific *Cflip*-deficient mice using the same strategy. These mice displayed an increase of circulating neutrophils and exhibited splenomegaly, which was due to the failure of macrophage differentiation and subsequent defective clearance of apoptotic neutrophils.

Huang *et al.* [93] also expressed *Cre* under *Itgax* promoter to delete *Cflip* specifically in dendritic cells (DCs). These mice developed spontaneous inflammatory arthritis, with an increase in autoreactive CD4^+^ T cells and the reduction of T regulatory cells. Very recently, Wu *et al.* [94] independently found that *Cflip*-deficient DCs generated by the same strategy displayed enhanced production of inflammatory cytokines upon innate signaling in a caspase-8 independent manner. These results indicated the unexpected suppressive roles of *Cflip* against innate immunity and inflammation signaling.

### 4.5. Conditional Knockout Mice Lacking cFLIP in Liver

Liver is the indispensable multi-functional organ for metabolic regulations, detoxifications, and the control of systemic homeostasis. Therefore, it is of great interest to determine whether *Cflip* is involved in the maintenance of healthy liver. Contrary to our expectation, liver-specific *Cflip*-deleted mice generated by the expression of *Cre* under *Alb* promoter (*Alb-Cre*) in *Cflip^F/F^* background were apparently healthy. However, this was due to the incomplete deletion of *Cflip* by *Alb-Cre* system, as we also reported that the complete ablation of *Cflip* in liver in mice expressing *Cre* using *Afp* enhancer-*Alb* promoter (*Alfp-Cre*) resulted in perinatal lethality [95]. Therefore, the mice generated with *Alb*-*Cre* system should be regarded as “*Cflip*-hypomorphic” rather than “*Cflip*-deficient”. Nevertheless, this animal model is useful to study the roles of *Cflip* under pathological conditions of adult liver. We and others reported that the reduction of *Cflip* increased the susceptibility of liver cells against various insults including anti-CD95 antibody, d-galactosamine and lipopolysaccharide, and concanavalin A [95,96]. Moreover, *Cflip* reduction resulted in the increased severity of acute liver injury and fibrosis induced by multiple chemical drugs, including carbon tetrachloride, thioacetamide, menadione, and streptozotocin [96,97,98,99]. In a different experimental setting, we crossed *Cflip^F/F^* mice with interferon-inducible *Mx1*-*Cre* transgenic mice, in which expression of *Cre* is induced in hepatocytes as well as hematopoietic cells after the administration of poly I:C. These mice also developed fatal hepatitis after poly I:C administration, thereby confirming the protecting role of *Cflip* in liver [95].

To achieve the systemic ubiquitous deletion of *Cflip* in adult mice, Gehrke *et al.* [110] expressed *ERT2-Cre* under *Rosa26* promoter in *Cflip^F/F^* mice. In these mice, intraperitoneal injection of tamoxifen resulted in the deletion of *Cflip* in multiple organs including liver, spleen and intestine, and these mice died within a few days after tamoxifen administration because of acute liver failure. This lethality was rescued by the transplantation of normal bone marrow or the depletion of macrophages, and the authors suggested that the observed acute liver failure was due to the activation of innate immune receptors.

### 4.6. Conditional Knockout Mice Lacking cFLIP in Epithelial Cells

Epithelial cells of intestine and skin are important barriers that protect animals from pathogenic organisms and toxic chemical compounds. We found that intestinal epithelium-specific deletion of *Cflip* generated by the expression *Cre* under *Vil1* promoter in *Cflip^F/F^* mice resulted in perinatal lethality [95]. Cell death by both apoptosis and necroptosis were already observed in intestinal epithelial cells (IEC) *in utero*, indicating that the observed defects were due to enhanced apoptosis of IEC rather than by pathogenic bacterial infection. Interestingly, additional deletion of *Tnfrsf1a* rescued the perinatal lethality of IEC-specific *Cflip*-deficient mice. These results were confirmed by Wittkopf *et al.* [101], who also showed that the acute ablation of *Cflip* in IEC of adult mice, generated by the tamoxifen treatment of *Cflip^F/F^* mice expressing *ERT2-Cre* under *Vil1* promoter, caused excessive apoptosis and gut inflammation.

For the specific deletion of *Cflip* in the epidermis, Weinlich *et al.* [102] locally administrated tamoxifen in the skin of *Cflip^F/F^* mice expressing *ERT2-Cre* in the whole body under *Rosa26* promoter. Panayotova-Dimitrova *et al.* [103] first generated epidermis-specific *Cflip*-deficient mice by expressing *Cre* under *Krt14* promoter. As these mice were embryonic lethal, the researchers then expressed *ERT2-Cre* instead of *Cre*, and carried out a similar experiment to delete *Cflip* by local tamoxifen treatment in skin. Both groups showed that *Cflip*-deleted skin displayed a severe inflammation and TNF-α-mediated caspase activation, which was rescued by antagonizing TNF-α. Importantly, this phenotype was not rescued by the additional *Ripk3* deletion [102], and necroptosis was not observed in *Cflip*-deficient skin [103]. These results suggest that the main function of *Cflip* in the epidermis is to prevent TNF-α-dependent apoptosis. 

### 4.7. cFLIP Isoform-Specific Transgenic Mice

The original floxed allele established by Zhang and He targeted the exon 1 of *Cflar* gene shared by both cFLIP_L_ and cFLIP_R_ (please remind that cFLIP_S_ is absent in mice). Therefore, *Cre*-mediated recombination resulted in the deletion of both cFLIP_L_ and cFLIP_R_. To identify the isoform-specific roles of cFLIP, BAC transgene-mediated expression of cFLIP_R_ into *Cflip*-deficient mice resulted in the generation of mice lacking only cFLIP_L_, thereby enabling the investigation of isoform-specific roles of cFLIP. T cells from mice lacking only cFLIP_L_ exhibited defective TCR-mediated proliferation and severely impaired effector T cell development after *Listeria monocytogenes* infection *in vivo*, although NF-κB activation was normal [104]. In addition, cFLIP_L_-deficient T cells underwent both apoptosis and RIPK1-mediated necroptosis upon TCR stimulation. Moreover, these cells also exhibited enhanced autophagy as a cytoprotective response [105].

As described in the previous section, it is somewhat controversial whether the physiological functions of cFLIP isoforms are similar or distinct. To clarify these issues, several isoform-specific transgenic mice were generated and analyzed, especially in T cells. Lens *et al.* [106] generated transgenic mice overexpressing cFLIP_L_ in T cells under *Hbb* promoter-human *CD2* enhancer. Tai *et al.* [107] used both *Cd2* and *Lck* promoter to express cFLIP_L_ in T cells. In contrast, Oehme *et al.* [116] generated transgenic mice expressing human cFLIP_S_ in T cells under *Lck* promoter. These transgenic T cells were all resistant to CD95-induced apoptosis, whereas activation-induced cell death was not affected. In this condition, mouse cFLIP_L_ and human cFLIP_S_ appeared to play similar roles. Increased activation of caspase-8 and NF-κB was observed in cFLIP_L_-transgenic CD8^+^ T cells, presumably due to increased caspase-8-cFLIP_L_ heterodimer formation. The authors suggested that a sub-lethal level of caspase-8 activity was required for T cell activation [108,109]. Wu *et al.* [110] reported that T-cell specific cFLIP_L_-transgenic mice produced reduced amount of IFN-γ and increased amounts of Th2 cytokines, and displayed Th2-driven enhanced sensitivity to OVA-induced asthma. Tseveleki *et al.* [111] reported that transgenic mice expressing human cFLIP_L_ in T cells under human *CD2* promoter showed augmented Th2 responses, and were resistant to Th1-driven experimental autoimmune encephalomyelitis. However, upon *Leishmania major* infection, these mice were able to overcome this Th2 bias and mounted a robust Th1 response to clear infection [112].

In a different situation, the roles of cFLIP_L_ and cFLIP_S_ were completely opposite. The increased T cell signaling in cFLIP_L_ transgenic mice reduced the severity of myocarditis induced by Coxsackievirus B3 (CVB3) infection [113]. In contrast, human cFLIP_S_ transgenic mice were highly sensitive to CVB3 infection and manifested increased severity of myocarditis [117]. However, disease pathologies may depend on multiple factors including genetic background, as T cell-specific cFLIP_L_ expression caused lupus-like syndrome in BALB/C but not in C57BL/6 strain [114].

In addition to T cells, several other tissues and organs were examined for the effect of cFLIP transgenic overexpression. In neurons, knockdown of cFLIP_L_ enhanced cell death induced by glucose-deprivation, whereas transgenic mice expressing cFLIP_L_ under *Nefl* promoter showed reduced lesion volume after permanent middle cerebral artery occlusion [118]. In thyroid epithelial cells, expression of cFLIP_L_ under rat *Tg* promoter protected cells from Fas-mediated apoptosis [119], and these transgenic mice were apparently healthy [120]. When granulomatous experimental autoimmune thyroiditis (G-EAT) was experimentally induced by the intravenous injection of mouse thyroglobulin, thyroid epithelial cell-specific cFLIP_L_ transgenic mice displayed better resolution of G-EAT, although disease severity were different between DBA/1 and CBA/J mice [121,122]. When G-EAT was induced in mice expressing cFLIP_L_ in T and B cells under *Hbb* promoter-human *CD2* enhancer to use as donor mice, these mice unexpectedly transferred less severe G-EAT to recipient mice due to reduced autoantibody production [115]. Moreover, heart-specific human cFLIP_L_ expression under *Myh6* promoter prevented cardiac remodeling induced by angiotensin II [123] and myocardial infarction [124]. Recently, Gordy *et al.* [125] used various tissue- and isoform-specific *Cflip*-deficient and transgenic mice to find that only cFLIP_L_ was necessary for mouse eosinophil survival in the presence of TNF-α both *in vitro* and *in vivo*. Collectively, these data suggest that cFLIP_L_ overexpression can prevent various tissues from pathological damages.

However, transgene-mediated overexpression of cFLIP isoforms may not always maintain homeostasis of tissue, organ, and whole body. When cFLIP_L_ was overexpressed selectively in testis under *Stra8* promoter [132], the male mice showed testis atrophy and reduced sperm motility [126]. This promoter also unexpectedly induced cFLIP_L_ overexpression in heart and skeletal muscle. Overexpression of cFLIP_L_ in heart reduced cardiac hypertrophy in response to pressure overload [127]. However, cFLIP_L_ overexpression in skeletal muscle resulted in muscle aging due to enhanced proliferation and concomitant apoptosis of muscle satellite cells [128]. Telieps *et al.* [129] generated transgenic mice expressing cFLIP_R_ specifically in hematopoietic cells under *Vav1* promoter. When challenged with *Listeria monocytogenes*, cFLIP_R_ transgenic mice showed better bacterial clearance than wild type mice, but developed a systemic lupus erythematosus-like autoimmunity with age [130]. These results infer that too much cell survival may be harmful to maintaining tissue homeostasis, and that the concentration and balance of cFLIP isoforms are critical determinants.

Mice do not express cFLIP_S_, whereas a single SNP determines whether *CFLAR* gene produces cFLIP_S_ or cFLIP_R_ in humans [13]. This may raise a question whether the absence of cFLIP_S_ in mice has any biological significance. The data from transgenic mice studies suggest that human cFLIP_S_ can functionally replace mouse cFLIP_R_. However, the extensive biochemical analysis revealed different biochemical properties between cFLIP_S_ and cFLIP_R_ [12]. Moreover, cFLIP_R_ expression was associated with lymphoma risk [13] and triptolide chemosensitivity [133]. Therefore, when expressed under natural promoter, cFLIP_S_ and cFLIP_R_ may cause different outcomes not observed in transgenic studies.

## 5. Conclusions

As we summarize in this review, cFLIP is a critical switch to control cell survival and death in various tissues. Most notably in TNF-α-mediated signaling pathway, cFLIP regulates both extrinsic apoptosis pathway and necroptosis pathway. From the clinical view, apoptosis induction is expected to be an effective way to kill tumor cells for cancer treatment. Indeed, a number of studies have indicated that the overexpression of cFLIP in various types of cancer cells may confer resistance against anti-cancer drugs [134]. In addition, blocking TNF-α signaling is a promising therapeutic strategy against several inflammatory diseases mediated by TNF-α. For example, TNF-neutralizing antibodies are approved and widely used for the treatment of various inflammatory diseases including rheumatoid arthritis, psoriatic arthritis, and Crohn’s disease [135]. Therefore, cFLIP has been regarded as an attractive target molecule to treat cancer and inflammation diseases. However, recent studies from gene-manipulated mice have also revealed the essential role of cFLIP in maintaining homeostasis in various tissues. Therefore, simple whole-body inhibition of cFLIP may cause unexpected side effects and reduce quality of life. As all cFLIP isoforms are intracellular proteins, antibody-mediated targeting of cFLIP may not be applicable. Therefore, therapeutic strategies may include the development of chemical compounds that modulate the function and stability of cFLIP. Moreover, numerous cellular studies have identified several drugs and chemical agents that inhibit the transcription of cFLIP, although their specificities remain to be evaluated. Further studies will be necessary for the complete understanding of cFLIP function and for the development of cFLIP-mediated therapeutic strategies without side effects.
